# 4-Bromo-1*H*-pyrrole-2-carb­oxy­lic acid

**DOI:** 10.1107/S1600536812034800

**Published:** 2012-08-11

**Authors:** Le Zheng, Fang Hu, Xiang Chao Zeng, Kai Ping Li

**Affiliations:** a Department of Chemistry, Jinan University, Guangzhou, Guangdong 510632, People’s Republic of China

## Abstract

In the title compound, C_5_H_4_BrNO_2_, the non-H atoms of the pyrrole ring and the Br atom are approximately coplanar, with an r.m.s. deviation from the best fit plane of 0.025 (6) Å;. The dihedral angle between the plane of the carb­oxy group and this plane is 14.1 (2)°. In the crystal, O—H⋯O hydrogen bonds link the mol­ecules together, forming corrugated sheets parallel to the *bc* plane.

## Related literature

For pyrrole compounds obtained from marine organisms, see: Liu *et al.* (2005 ▶); Faulkner (2002 ▶). For the bioactivity of pyrrole derivatives, see: Banwell *et al.* (2006 ▶); Sosa *et al.* (2002 ▶). For related structures, see: Zeng *et al.* (2007 ▶); Tang *et al.* (2008 ▶).
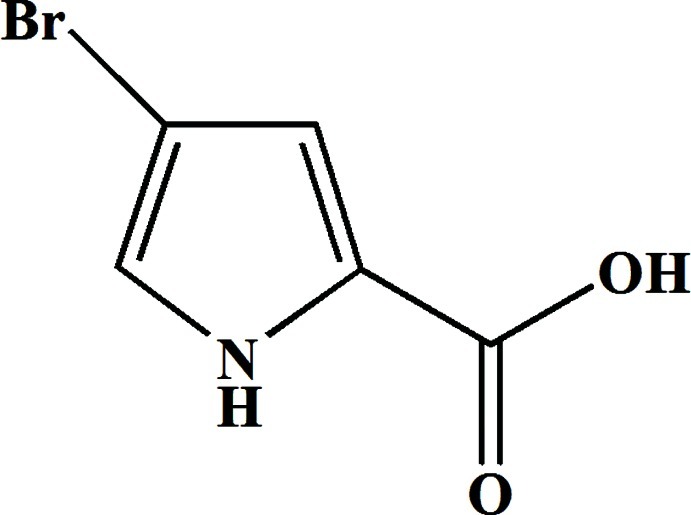


## Experimental

### 

#### Crystal data

C_5_H_4_BrNO_2_
*M* *_r_* = 190.00
Monoclinic, 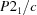

*a* = 16.0028 (13) Å
*b* = 4.9046 (6) Å
*c* = 8.2367 (7) Å
β = 93.199 (7)°
*V* = 645.47 (11) Å^3^
*Z* = 4
Mo *K*α radiation
μ = 6.29 mm^−1^
*T* = 293 K
0.24 × 0.20 × 0.14 mm

#### Data collection

Oxford Gemini S Ultra area-detector diffractometer
Absorption correction: multi-scan (*CrysAlis PRO*; Oxford Diffraction, 2010 ▶) *T*_min_ = 0.314, *T*_max_ = 0.473
2436 measured reflections
1387 independent reflections
1081 reflections with *I* > 2σ(*I*)
*R*_int_ = 0.021

#### Refinement

*R*[*F* ^2^ > 2σ(*F* ^2^)] = 0.034
*wR*(*F* ^2^) = 0.081
*S* = 1.10
1387 reflections
85 parameters
H-atom parameters constrained
Δρ_max_ = 0.39 e Å^−3^
Δρ_min_ = −0.45 e Å^−3^

### 

Data collection: *CrysAlis PRO* (Oxford Diffraction, 2010 ▶); cell refinement: *CrysAlis PRO*; data reduction: *CrysAlis PRO*; program(s) used to solve structure: *SHELXS97* (Sheldrick, 2008 ▶); program(s) used to refine structure: *SHELXL97* (Sheldrick, 2008 ▶); molecular graphics: *SHELXTL* (Sheldrick, 2008 ▶); software used to prepare material for publication: *SHELXTL*.

## Supplementary Material

Crystal structure: contains datablock(s) I, global. DOI: 10.1107/S1600536812034800/fj2579sup1.cif

Structure factors: contains datablock(s) I. DOI: 10.1107/S1600536812034800/fj2579Isup2.hkl

Supplementary material file. DOI: 10.1107/S1600536812034800/fj2579Isup3.cml

Additional supplementary materials:  crystallographic information; 3D view; checkCIF report

## Figures and Tables

**Table 1:** Hydrogen-bond geometry (Å, °)

*D*—H⋯*A*	*D*—H	H⋯*A*	*D*⋯*A*	*D*—H⋯*A*
O2—H2′⋯O1^i^	1.07	1.86	2.914 (4)	166
O2—H2⋯O1^ii^	0.82	2.28	3.030 (4)	153

Symmetry codes: (i) 
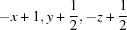
; (ii) 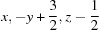.

**Fig. 1.**
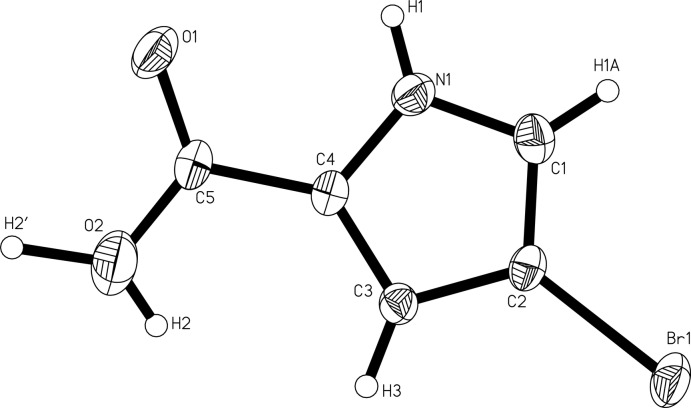
The molecular structure of the title compound, with the atom-numbering scheme. Displacement ellipsoids are drawn at the 30% probability level.

**Fig. 2.**
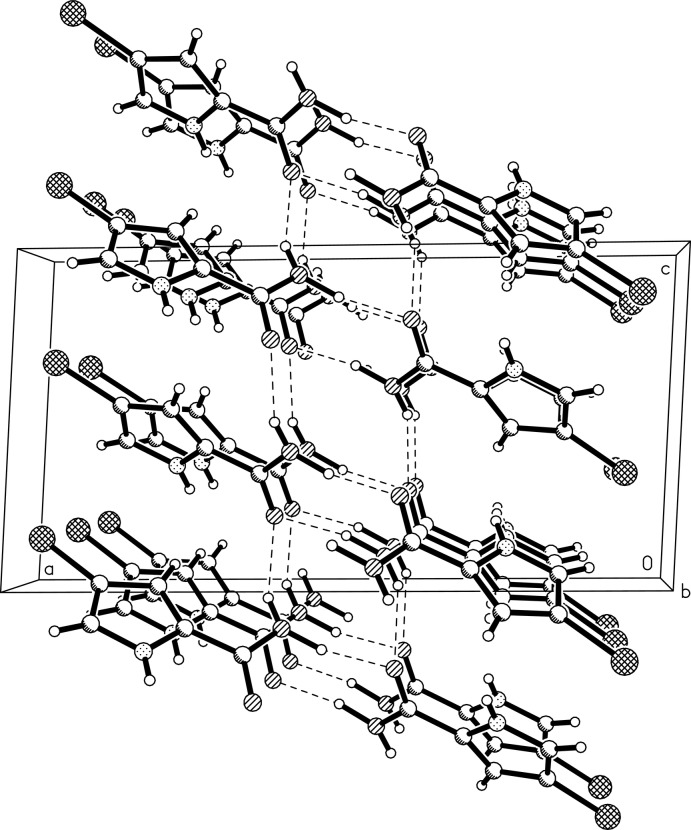
The crystal packing. Dashed lines indicate hydrogen bonds.

**Fig. 3.**
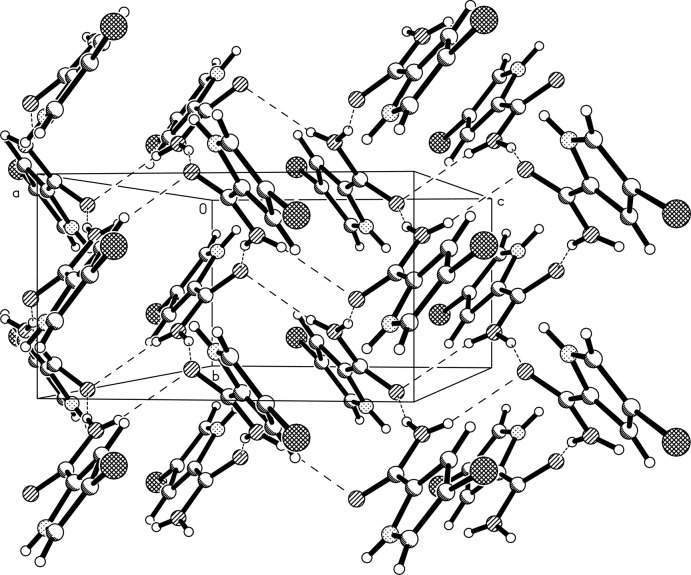
sheet formed by weak hydrogen bonds (dashed lines).

## supplementary crystallographic information

### Comment

Pyrrole derivatives are well known in many marine organisms (Faulkner, 2002), some show important bioactivities, such as antitumor activity (Banwell *et al.*, 2006) and protein kinase inhibiting activity (Sosa *et al.*, 2002). These are the reasons why they have attracted our interest. This study is relevant to our previous studies on Methyl 2-(4,5-dibromo-1*H*-pyrrole-2-carboxamido)propionate (Zeng *et al.*, 2007) and 1*H*-Pyrrole-2-carboxylic acid (Tang *et al.*, 2008).

In the title molecule, bond lengths and angles are unexceptional. The non-H atoms of the pyrrole ring and Br atom are approximately coplanar (plane 1), with r.m.s. deviation from the best fit plane of 0.025 (6)°, the dihedral angle between the carboxy plane and Plane 1 is 14.1 (2)°. The OH hydrogen atom is disordered over two positions (shown as in Fig. 1), which form weak intermolecular O2—H2···O1 hydrogen bonds respectively (Table 1). In the crystal, the above hydrogen bonds link the molecules into corrugated sheets parallel to the *bc* plane (shown as in Fig. 2 and Fig. 3).

### Experimental

The methyl 4-bromopyrrole-2-carbonylaminoacetate (1.30 g, 5 mmol) and potassium carbonate (1.38 g, 10 mmol) were added to acetonitrile (20 ml), the mixture was stirred at reflux for 24 h. After the reaction mixture was cooled and filtered, the filtrate was evaporated *in vacuo*, and then the residue was chromatographed over Si gel 60 using EtOAc-petroleum ether (1:2.5) as eluting solvent and the title compound (I) was obtained (55.2% yield). Light yellow monoclinic crystals suitable for X-ray analysis (m.p. 424 K) grew over a period of one week when the EtOH solution of I was exposed to the air at room temperature.

### Refinement

All non-H atoms were refined with anisotropic displacement parameters. The H atoms except the H(OH) were positioned geometrically[C—H = 0.93 Å for CH, and N—H = 0.86 Å] and refined using a riding model, with *U*_iso_ = 1.2*U*_eq_ of the parent atom. The H atoms attached to hydroxy O atoms were found automatically.

### Crystal data

**Table:**

C_5_H_4_BrNO_2_	*F*(000) = 368
*M**_r_* = 190.00	*D*_x_ = 1.955 Mg m^−^^3^
Monoclinic, *P*2_1_/*c*	Melting point < 424 K
Hall symbol: -P 2ybc	Mo *K*α radiation, λ = 0.71073 Å
*a* = 16.0028 (13) Å	Cell parameters from 950 reflections
*b* = 4.9046 (6) Å	θ = 3.5–29.1°
*c* = 8.2367 (7) Å	µ = 6.29 mm^−^^1^
β = 93.199 (7)°	*T* = 293 K
*V* = 645.47 (11) Å^3^	Block, light yellow
*Z* = 4	0.24 × 0.20 × 0.14 mm

### Data collection

**Table:**

Oxford Gemini S Ultra area-detector diffractometer	1387 independent reflections
Radiation source: fine-focus sealed tube	1081 reflections with *I* > 2σ(*I*)
Graphite monochromator	*R*_int_ = 0.021
φ and ω scans	θ_max_ = 27.0°, θ_min_ = 3.8°
Absorption correction: multi-scan (*CrysAlis PRO*; Oxford Diffraction, 2010)	*h* = −20→12
*T*_min_ = 0.314, *T*_max_ = 0.473	*k* = −5→6
2436 measured reflections	*l* = −10→10

### Refinement

**Table:**

Refinement on *F*^2^	Primary atom site location: structure-invariant direct methods
Least-squares matrix: full	Secondary atom site location: difference Fourier map
*R*[*F*^2^ > 2σ(*F*^2^)] = 0.034	Hydrogen site location: inferred from neighbouring sites
*wR*(*F*^2^) = 0.081	H-atom parameters constrained
*S* = 1.10	*w* = 1/[σ^2^(*F*_o_^2^) + (0.034*P*)^2^ + 0.2705*P*] where *P* = (*F*_o_^2^ + 2*F*_c_^2^)/3
1387 reflections	(Δ/σ)_max_ < 0.001
85 parameters	Δρ_max_ = 0.39 e Å^−^^3^
0 restraints	Δρ_min_ = −0.45 e Å^−^^3^

### Special details

**Table:**

Geometry. All e.s.d.'s (except the e.s.d. in the dihedral angle between two l.s. planes) are estimated using the full covariance matrix. The cell e.s.d.'s are taken into account individually in the estimation of e.s.d.'s in distances, angles and torsion angles; correlations between e.s.d.'s in cell parameters are only used when they are defined by crystal symmetry. An approximate (isotropic) treatment of cell e.s.d.'s is used for estimating e.s.d.'s involving l.s. planes.
Refinement. Refinement of *F*^2^ against ALL reflections. The weighted *R*-factor *wR* and goodness of fit *S* are based on *F*^2^, conventional *R*-factors *R* are based on *F*, with *F* set to zero for negative *F*^2^. The threshold expression of *F*^2^ > σ(*F*^2^) is used only for calculating *R*-factors(gt) *etc*. and is not relevant to the choice of reflections for refinement. *R*-factors based on *F*^2^ are statistically about twice as large as those based on *F*, and *R*- factors based on ALL data will be even larger.

### Fractional atomic coordinates and isotropic or equivalent isotropic displacement parameters (Å^2^)

**Table:**

	*x*	*y*	*z*	*U*_iso_*/*U*_eq_	Occ. (<1)
Br1	0.07690 (2)	0.68153 (9)	−0.16395 (4)	0.04763 (17)	
N1	0.24925 (19)	0.3185 (6)	0.1354 (4)	0.0400 (7)	
H1	0.2653	0.1983	0.2065	0.048*	
C3	0.2505 (2)	0.6756 (8)	−0.0297 (4)	0.0321 (7)	
H3	0.2674	0.8285	−0.0861	0.039*	
O1	0.40382 (15)	0.4281 (6)	0.2816 (3)	0.0514 (7)	
C4	0.29799 (19)	0.5248 (7)	0.0817 (4)	0.0321 (8)	
C2	0.17094 (19)	0.5519 (8)	−0.0414 (4)	0.0327 (8)	
O2	0.4311 (2)	0.7498 (8)	0.0940 (4)	0.0731 (10)	
H2	0.4079	0.8099	0.0102	0.013 (15)*	0.50
H2'	0.4898	0.7973	0.1562	0.009 (13)*	0.50
C1	0.1716 (2)	0.3316 (8)	0.0598 (5)	0.0421 (9)	
H1A	0.1274	0.2128	0.0743	0.050*	
C5	0.3821 (2)	0.5608 (9)	0.1583 (4)	0.0373 (8)	

### Atomic displacement parameters (Å^2^)

**Table:**

	*U*^11^	*U*^22^	*U*^33^	*U*^12^	*U*^13^	*U*^23^
Br1	0.0310 (2)	0.0638 (3)	0.0466 (2)	0.00130 (19)	−0.01071 (15)	0.0010 (2)
N1	0.0398 (17)	0.0338 (17)	0.0453 (16)	0.0004 (15)	−0.0077 (13)	0.0041 (15)
C3	0.0309 (17)	0.037 (2)	0.0284 (15)	−0.0035 (16)	−0.0004 (12)	0.0005 (16)
O1	0.0376 (14)	0.0662 (19)	0.0487 (14)	0.0089 (14)	−0.0119 (11)	0.0072 (15)
C4	0.0274 (15)	0.036 (2)	0.0329 (15)	0.0019 (15)	−0.0013 (12)	−0.0050 (16)
C2	0.0279 (16)	0.041 (2)	0.0289 (15)	0.0017 (16)	−0.0025 (12)	−0.0053 (17)
O2	0.052 (2)	0.097 (3)	0.069 (2)	−0.0130 (18)	−0.0158 (16)	0.004 (2)
C1	0.0357 (19)	0.042 (2)	0.048 (2)	−0.0074 (17)	−0.0006 (16)	−0.0045 (19)
C5	0.0278 (17)	0.048 (2)	0.0357 (17)	0.0020 (18)	−0.0038 (13)	−0.0053 (19)

### Geometric parameters (Å, º)

**Table:**

Br1—C2	1.876 (3)	O1—C5	1.239 (4)
N1—C1	1.360 (5)	C4—C5	1.465 (4)
N1—C4	1.366 (4)	C2—C1	1.364 (5)
N1—H1	0.8600	O2—C5	1.342 (5)
C3—C4	1.375 (5)	O2—H2	0.8200
C3—C2	1.408 (4)	O2—H2'	1.0702
C3—H3	0.9300	C1—H1A	0.9300
			
C1—N1—C4	109.9 (3)	C3—C2—Br1	125.8 (3)
C1—N1—H1	125.1	C5—O2—H2	109.5
C4—N1—H1	125.1	C5—O2—H2'	118.5
C4—C3—C2	106.1 (3)	H2—O2—H2'	132.0
C4—C3—H3	126.9	N1—C1—C2	107.1 (3)
C2—C3—H3	126.9	N1—C1—H1A	126.5
N1—C4—C3	108.1 (3)	C2—C1—H1A	126.5
N1—C4—C5	118.5 (3)	O1—C5—O2	122.9 (3)
C3—C4—C5	133.1 (3)	O1—C5—C4	119.9 (3)
C1—C2—C3	108.8 (3)	O2—C5—C4	117.1 (3)
C1—C2—Br1	125.2 (3)		
			
C1—N1—C4—C3	0.6 (4)	C3—C2—C1—N1	0.8 (4)
C1—N1—C4—C5	175.0 (3)	Br1—C2—C1—N1	−175.1 (2)
C2—C3—C4—N1	−0.1 (4)	N1—C4—C5—O1	−8.7 (5)
C2—C3—C4—C5	−173.3 (3)	C3—C4—C5—O1	163.9 (4)
C4—C3—C2—C1	−0.5 (4)	N1—C4—C5—O2	174.5 (3)
C4—C3—C2—Br1	175.4 (2)	C3—C4—C5—O2	−12.8 (6)
C4—N1—C1—C2	−0.9 (4)		

### Hydrogen-bond geometry (Å, º)

**Table:**

*D*—H···*A*	*D*—H	H···*A*	*D*···*A*	*D*—H···*A*
O2—H2′···O1^i^	1.07	1.86	2.914 (4)	166
O2—H2···O1^ii^	0.82	2.28	3.030 (4)	153

Symmetry codes: (i) −*x*+1, *y*+1/2, −*z*+1/2; (ii) *x*, −*y*+3/2, *z*−1/2.
